# Magnesium Sulfate as a Second-Line Tocolytic Agent for Preterm Labor: A Randomized Controlled Trial in Kyushu Island

**DOI:** 10.1155/2011/965060

**Published:** 2011-06-16

**Authors:** Yasuyuki Kawagoe, Hiroshi Sameshima, Tsuyomu Ikenoue, Ichiro Yasuhi, Tatsuhiko Kawarabayashi

**Affiliations:** ^1^Department of Obstetrics and Gynecology, University of Miyazaki, 5200 Kihara, Kiyotake, Miyazaki 889-1692, Japan; ^2^Department of Obstetrics and Gynecology, National Hospital Organization Nagasaki Medical Center, Nagasaki 856-8562, Japan; ^3^Department of Obstetrics and Gynecology, Fukuoka University, Fukuoka Department of Obstetrics and Gynecology, Fukuoka University, Fukuoka 814-0180, Japan

## Abstract

*Objectives*. We evaluated the efficacy of magnesium sulfate as a second-line tocolysis for 48 hours. *Materials and Methods*. A multi-institutional, simple 2-arm randomized controlled trial was performed. Forty-five women at 22 to 34 weeks of gestation were eligible, whose ritodrine did not sufficiently inhibit uterine contractions. After excluding 12 women, 33 were randomly assigned to either magnesium alone or combination (ritodrine and magnesium). The treatment was determined as effective if the frequency of uterine contraction was reduced by 30% at 48 hours of the treatment. *Results*. After magnesium sulfate infusion, 90% prolonged their pregnancy for >48 hours. Combination therapy was effective in 95% (18/19), which was significantly higher than 50% (7/14) for magnesium alone. *Conclusion*. This randomized trial revealed that combination therapy significantly reduced uterine contractions, suggesting that adjuvant magnesium with ritodrine is recommended, rather than changing into magnesium alone, when uterine contractions are intractable with ritodrine infusion.

## 1. Introduction

Acute tocolysis prevents preterm labor for 48 hours, which is the critical period for antenatal steroid administration or maternal transfer to perinatal centers to improve neonatal outcomes. Evidence supporting the effectiveness of magnesium sulfate for tocolysis is currently being questioned. Meta-analysis comparing magnesium sulfate and either placebo, no treatment, or alternative tocolytic agents showed that magnesium reduces the risk of birth within 48 hours by 15%, which is not statistically significant (relative risk (RR) 0.85, 95% confidence interval (CI) 0.58 to 1.25) [1]. A more recent report evaluating 19 randomized controlled trials reveals that magnesium fails to reduce delivery rate within 48 hours [2]. However, magnesium sulfate is currently the most commonly used tocolytic agent in America. 

In 2008, the Ministry of Health, Labor and Welfare of Japan finally approved magnesium sulfate as a tocolytic agent, but its use is restricted to the second-line tocolysis when the effect of a betamimetic agonist is not sufficient to reduce uterine contractions. Here, a question arises as to how we administer magnesium sulfate to these women, that is, to stop the betamimetic agonist and change into magnesium sulfate or add magnesium sulfate to the betamimetic agonist. The efficacy of tocolysis when using a combination of a betamimetic agonist and magnesium sulfate has been investigated, and some retrospective studies have shown that a combination therapy may be effective in prolonging gestation [3–7]. On the other hand, a prospective randomized study showed that such treatment may not improve tocolytic efficacy [8] but produced an unacceptable increase in serious maternal side effects. At present, there are no randomized controlled trials comparing the 2 modes of using magnesium sulfate, either change or addition, as a second-line tocolytic agent in cases where a betamimetic agonist alone is not sufficient to inhibit preterm labor. Since the mechanisms of tocolytic action differ between magnesium sulfate and betamimetics, we hypothesized that adding magnesium sulfate to the treatment would be superior to using magnesium sulfate alone for the inhibition of uterine contractions. We tested this hypothesis in a prospective, randomized controlled trial.

## 2. Materials and Methods

This study was approved by the institutional review board of the University of Miyazaki and all the participating institutions. A multi-institutional, prospectively randomized trial was performed from April 2007 to March 2009. We collected data from 14 institutions: 3 were university hospitals and the remaining 11 were regional referral centers in Kyushu, Japan. Informed consent was obtained in each institution. 

Inclusion criteria were singleton or dichorionic twin pregnancies with intact membranes, showing clinical signs of preterm labor at 22 to 34 weeks of gestation. The starting point of 22 weeks of gestation was chosen because their survival rate surpasses more than 50% in Japan, and they are legally protected for the chance of survival in Japan. Definition of preterm labor in Japan is different from that of others, that is, as the presence of sensitive uterine contractions (6/hour or more) with the duration of >30 seconds, and changes in cervical dilation or cervical length are not taken into consideration. Dating of pregnancy was confirmed by first-trimester ultrasound fetometry. 

Exclusions were women with clinical intrauterine infection, abruption, cervical dilatation >5 cm, medical complications contraindicating tocolysis, and evidence of nonreassuring fetal status. Clinical intrauterine infection was diagnosed according to criteria previously reported [9]: body temperature >38°C, maternal tachycardia >100 bpm, uterine tenderness, odor discharge, and leukocytosis >15,000/microliter. 

### 2.1. Study Protocol and Treatment

Since magnesium sulfate is to be used for second-line tocolysis when a betamimetic agonist is not sufficient to reduce uterine contractions, all women received a first-line treatment (ritodrine) before the start of magnesium sulfate. Ritodrine infusion was titrated in the decided manner with a starting dose of 50 micrograms/min and an increment of 50 micrograms/min over 30 to 60 minutes, until the maximum dose of 200 micrograms/min. When uterine contractions were intractable by its maximal dose, magnesium sulfate was given. 

**Figure 1:**
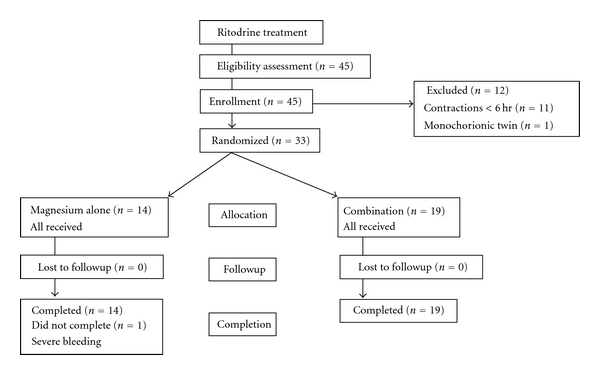
Flowchart of a simple 2-arm randomized controlled trial.

We applied a simple 2-arm protocol to add the magnesium sulfate infusion (Figure 1). One arm is to discontinue ritodrine and change into magnesium sulfate with a loading dose of 4 gm over 30 minutes followed by a maintenance dose of 1.0-2.0 gm/hr (magnesium alone), which was titrated as uterine contractions changed. When uterine contraction increased by magnesium alone, attending physicians could resume ritodrine at discretion. The other arm is to continue ritodrine and add magnesium sulfate with a maintenance dose of 1.0-2.0 gm/hr (combination). Both drugs were given with separate infusion pumps, and the total volume of intravenous infusion was limited to <1,000 mL per day. We did not have other protocol arms such as placebo or no treatment, because these treatments were considered unethical by our ethics committee for women having intractable uterine contractions even with the maximum dose of ritodrine. After 48 hours of the allocated treatments, the physicians chose either to stop or to continue tocolysis until delivery.

### 2.2. Sample Size

Previous retrospective reports showed that 60–90% of women given the combined treatment of magnesium sulfate and ritodrine experienced a prolonged pregnancy for ≥48 hours [3–7]. Thus, we assumed that the inhibition rate to decrease uterine contraction using the combination treatment was 20% in this study. Similarly, the inhibition rate for magnesium alone was estimated as 45%. When the efficacy was compared using *z*-statistics with a level of significance of *α* = 0.05 (one-tailed) and a power of 80%, the required number of women was calculated to be 42 women per group.

### 2.3. Randomization

We applied a block randomization method. The block random size was arbitrarily chosen as 6, which mathematically requires 14 institutions. Among the 14 institutions, 7 were designed to start magnesium alone for the first 3 women, followed by the combination therapy for the next 3 women. The remaining 7 institutions were to conduct treatments in the reverse manner. 

 As mentioned above, all the 14 institutions had the same inclusion criteria for preterm labor and the same treatment protocol for the first-line tocolysis. Similarly, Cesarean delivery was basically chosen for premature breech, nonvertex twins, and previous cesarean birth, and antenatal steroid was administered only once when delivery was impending. 

### 2.4. Evaluation

The number of uterine contractions was assessed by cardiotocogram 48 hours after initiation of magnesium sulfate. Treatment was defined as effective if uterine contractions were reduced by more than 30% in frequency compared to those occurring during the last 2 hours before magnesium sulfate infusion. Treatment was defined as ineffective if uterine contractions were not reduced by 30%, labor progressed with apparent changes in cervical findings, or the attending physician resumed ritodrine in the magnesium-alone group due to insufficient tocolytic efficacy. For the intention-to-treat analysis, women who needed to deliver before 48 hours of observation due to some complications other than preterm labor were also classified as ineffective.

### 2.5. Statistics

Entry characteristics and outcome variables were analyzed using the Mann-Whitney *U* test, the unpaired *t*-test, and chi-square analysis with the Fisher exact test. Multiple logistic regression analysis was performed to obtain relative risk (RR) and the 95% confidence interval (95% CI) for the combination treatment, where an effective treatment was assigned the value 1 and an ineffective treatment the value 0. *P* values <.05 are considered statistically significant. Data are expressed as mean ± standard deviation.

## 3. Results and Discussion

After 2 years of data collection, we had 45 eligible women (Figure 1), but 12 women were excluded because they failed to meet the inclusion criteria of preterm labor (*n* = 11), and one was excluded due to monochorionic twin. The remaining 33 women remained for analysis, which was less than the required sample size (*n* = 84). Our control center decided to extend the study period for 6 months, but no further eligible case was enrolled. Thus, we decided to quit sample collection and to perform analyses to determine if there were some significant results.

Maternal demographic and clinical characteristics at randomization were similar between the 2 groups (Table 1) regarding maternal age, parity, gestational age at admission, and incidence of prior preterm birth. Dilatation of the uterine cervix was less than 3 cm in all women.

Timing of magnesium sulfate infusion varied from <24 hours to 30 days after commencing ritodrine tocolysis. The prevalence of women who required second-line magnesium treatment within 24 hours of ritodrine tocolysis was 12% (4/33) in total, 7% (1/14) for magnesium alone, and 16% (3/19) for the combination treatment (not significant, Fisher test). 

Obstetric characteristics were also not statistically different (Table 2). Delivery rate <48 hours of the allocated treatment was not significantly different between the 2 groups. Similarly, delivery week and cesarean delivery rate were not significantly different. 

**Figure 2:**
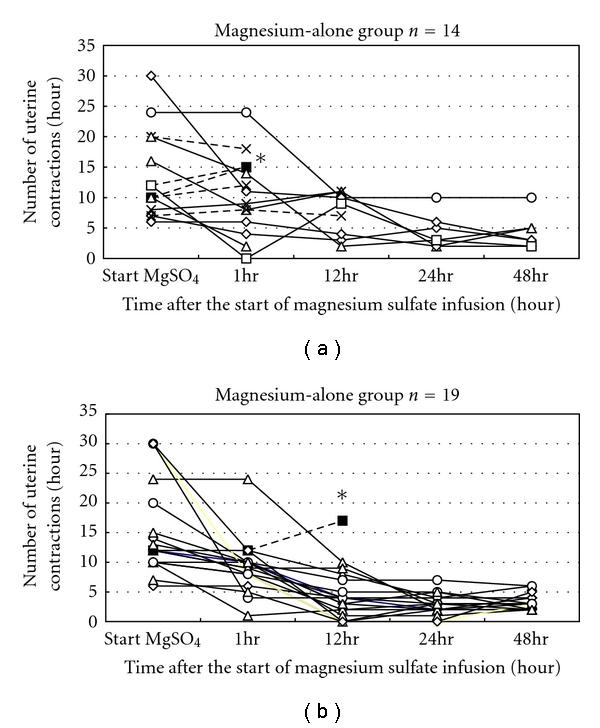
Temporal changes in uterine contractions after the start of magnesium sulfate treatment in the magnesium-alone group (a) and combination group (b). The dotted lines in the magnesium-alone group represent those who required reinfusion of ritodrine to inhibit uterine contraction. Asterisks represent those who delivered within 48 hours of magnesium infusion.

The changes in uterine contractions during the first 48 hours of magnesium infusion are depicted in Figure 2. The number of uterine contractions decreased as the duration of magnesium sulfate treatment increased. One woman in each group progressed and delivered vaginally within 12 hours, and thus treatment for these women was classified as ineffective. In the magnesium-alone group, uterine contractions did not decrease in 5 women, who subsequently required ritodrine supplementation during the first 48 hours. These 5 cases were also classified as ineffective. In the magnesium-alone group, another women delivered before 48 hours because of massive genital bleeding of unknown causes, which occurred after 12 hours of magnesium infusion without improvement of uterine contractions. This case was also classed as ineffective. 

Intention-to-treat analysis showed that the combination treatment significantly decreased the prevalence of ineffectiveness compared to magnesium-alone treatment (Table 3, *P* < .01 by Fisher test, RR; 0.06, 95% CI 0.006 to 0.54 by logistic regression analysis). In other words, the combination treatment is 15-fold more effective in inhibiting uterine contractions than magnesium alone. 

There were no serious maternal side effects such as pulmonary edema, respiratory complications, ileus-like symptoms, or rhabdomyolysis in both groups. Two women felt mild and transient muscle weakness within 48 hours of magnesium infusion (one for each treatment group), which resolved spontaneously.

At 48 hours of the allocated treatment, all physicians chose to continue tocolysis until delivery. Neonatal outcomes for both groups are listed in Table 4. All infants survived and did not suffer from neurological damage at 1 year of age or older. There was no significant difference in birth weight, gestational weeks at delivery, prolongation time after magnesium sulfate treatment, and incidence of low Apgar score between the 2 groups. 

In this randomized controlled trial, we found that 90% of women (29/33) delayed their delivery for >48 hours after magnesium sulfate infusion, when ritodrine alone failed to inhibit uterine contractions. We also found that the combination treatment was more effective than magnesium alone in decreasing uterine contractions.

Animal studies have shown that uterine activity is initially inhibited by intravenous infusion of ritodrine for 6 hours, but activity returns to the preinfusion level by 11 to 16 hours despite continuous infusion [10, 11]. Previous reports based on *in vitro* experiments also showed that continuous exposure of myometrium to betamimetic agonists resulted in an initial myometrial relaxation followed by desensitization and return of myometrial contractions [12–14]. In the present study, 90% (30/33) of women received magnesium sulfate after 24 hours of ritodrine infusion, suggesting that they became desensitized and uterine contractions resumed, which was inhibited by administering magnesium sulfate. 

Betamimetic agonists, on binding with beta-2 receptors on the cell membrane, activate adenlylate cyclase to increase the intracellular concentration of cyclic adenosine monophosphate, resulting in decreased availability of free intracellular calcium for actin and myosin, leading to relaxation of uterine smooth muscle. On the other hand, magnesium sulfate acts on the voltage-dependent calcium channels to competitively block the calcium influx, leading to the inhibition of calcium-dependent myosin light-chain kinase phosphorylation. It is logical to speculate that there may be some additive effects that inhibit uterine contractions since both agents have different mechanisms of action. Historically, the combination therapy may [4–7] or may not [8] improve tocolytic efficacy. Our results agreed with that a combination treatment is superior to magnesium alone in prolonging pregnancy, even in the stage of desensitization during ritodrine treatment. 

 We used magnesium sulfate for the second-line tocolysis when the betamimetic effect was not sufficient to reduce uterine contractions. This situation is unique because the fact that a betamimetic agonist alone failed to inhibit uterine contractions suggests that the situation is more likely to occur in true labor than false labor, and the placebo effect may not interfere with the results. Under these conditions, the adjunctive use of magnesium sulfate with ritodrine is 15-fold more effective in inhibiting uterine contractions than magnesium sulfate alone. 

This study has several advantages. One is its nature of a randomized controlled trial. Another is the above-mentioned study protocol where the placebo effect is taken into consideration. This study also has some limitations. The major problem is its underpowered number and uneven allocation due to incomplete block randomization. Only 3 of the 14 institutions fulfilled the allocated number (*n* = 6) of data collection, 3 finished the first allocated block (*n* = 3) with the second block halfway, and 2 institution did not enroll any women. The control center strongly requested for enrollment and extended the study period for 6 months, but no cases were added. Therefore, only 45 women were included, which is much smaller than the required number. Even with this major limitation, our conclusion is of clinical importance that combination of magnesium with ritodrine is 15-fold more effective to inhibit uterine contractions than magnesium alone, in which ritodrine alone is not sufficient to inhibit them. 

## 4. Conclusions

This randomized trial revealed that adjuvant magnesium with ritodrine significantly reduced uterine contractions than magnesium alone, when uterine contractions are intractable with ritodrine infusion.

## Figures and Tables

**Table 1:** Maternal demographic and clinical characteristics at randomization.

	Magnesium-alone group	Combination group	Comparison
No. of pregnancies	14	19	—
Twin gestations	1 (7%)	4 (21%)	NS
Maternal age (years)	31.3 ± 4.6	30.3 ± 4.8	NS
Nulliparous women	8 (57%)	8 (42%)	NS
Prior preterm birth	1 (7%)	3 (16%)	NS
Gestational age on admission (wk)	28.3 ± 4.4	27.2 ± 3.4	NS
Gestational age at start of ritodrine (wk)	28.1 ± 3.7	27.6 ± 3.2	NS
Gestational age at start of MgSO_4_ (wk)	29.3 ± 3.2	28.6 ± 3.4	NS
Cervical dilatation ≥3 cm	0/14	0/19	NS

NS; not significant. MgSO_4_; magnesium sulfate. Mean ± SD.

**Table 2:** Obstetric characteristics at the start of the allocated treatment.

	Magnesium-alone group (*n* = 14)	Combination group (*n* = 19)	Comparison
Delivery <48 hours after MgSO_4_ infusion	2/14 (14%)	1/19 (5%)	NS
Delivery week (wk)	35.5 ± 2.2	32.8 ± 4.6	NS
Cesarean delivery (%)	8/14 (57%)	9/19 (47%)	NS

NS; not significant. Mean SD. Comparisons were performed by Fisher test or unpaired *t*-test.

**Table 3:** Changes in uterine contractions at 48 hours after magnesium sulfate infusion.

	Magnesium-alone group (*n* = 14)	Combination group (*n* = 19)
Decreased	7 (50%)	18 (95%)
Unchanged ~ Increased	7 (50%)	1* (5%)

*P < .01, Fisher test.

**Table 4:** Neonatal outcomes of magnesium alone and combination groups.

	MgSO_4_ therapy group	Combined therapy group	Comparison
Number of infants	15	23	—
Birth weight (gm) (range)	2378 ± 478 (1704–3656)	1912 ± 681 (488–2876)	NS
Gestational age (wk) (range)	35.5 ± 2.2 (32–41)	32.8 ± 4.6 (24–38)	NS
Prolongation after magnesium sulfate therapy (days)	41.5 ± 32.3 (0–108)	27.8 ± 22.3 (2–59)	NS
Apgar score at 5 min (<6)	0/15	1*/23	NS

*Neonate delivered at 24 weeks gestations (birth weight 488 gm with Apgar score 2/5 (1 min/5 min)).

NS, not significant by unpaired *t*-test, Mann-Whitney *U* test, or Fisher test.
